# Case Report: Lidocaine Toxicity Presenting with Focal Neurologic Findings

**DOI:** 10.5811/cpcem.62132

**Published:** 2026-07-28

**Authors:** Timothy Yeung, Isaac Estrada, Tammy H. Phan, Emmelyn J. Samones, Sharmin Kalam

**Affiliations:** * Loma Linda University Medical Center, Department of Emergency Medicine, Loma Linda, California; † Loma Linda University Health, School of Medicine, Loma Linda, California

**Keywords:** lidocaine toxicity, focal neurologic findings, intralipid infusion, local anesthetic systemic toxicity (LAST), case report

## Abstract

**Introduction:**

Lidocaine toxicity is a rare but potentially dangerous consequence of a frequently used medication. Most commonly, it has generalized neurologic and cardiac effects. Discontinuation of lidocaine is sufficient with less severe symptoms. With more severe symptoms, such as seizures and arrhythmia, intravenous (IV) lipid emulsion therapy can be used. To date, lidocaine toxicity presenting as focal neurologic deficits has rarely been documented in the literature. In the case reported here, the patient was receiving lidocaine injections when she developed palpitations and left arm and facial weakness and numbness, which resolved after IV lipid emulsion therapy.

**Case Report:**

A 52-year-old female with a past medical history of migraines, cholecystectomy, nephrolithiasis, and hypothyroidism presented to the emergency department (ED) via ambulance from her dental office with acute neurologic symptoms. While receiving an injection of lidocaine intraorally at the dental office, she experienced palpitations and left arm and left facial weakness with numbness. She continued to have symptoms upon arrival to the ED. Given concern for acute stroke, she underwent imaging, which was negative for large vessel occlusions or hemorrhage. She was given IV lipid emulsion therapy for suspected lidocaine toxicity with resolution of symptoms. She was cleared by oral and maxillofacial surgery and discharged with outpatient follow-up.

**Conclusion:**

We identified a patient who developed neurologic symptoms during lidocaine administration, with no radiographic evidence for stroke. Given the timing of symptoms and lidocaine administration, we administered IV lipid emulsion therapy with resolution of the patient’s symptoms. Thus, we strongly suspect our patient had lidocaine toxicity with focal neurologic findings.

## INTRODUCTION

Lidocaine is used in nearly all fields of medicine, with uses ranging from local anesthesia for procedures and multimodal pain control for acute and chronic pain to antiarrhythmic therapy. It is metabolized by cytochrome P450 1A2 and cytochrome P450 3A4 in the liver and excreted via urine with a half-life of 1.5–2 hours.^1^ In plasma, lidocaine is primarily bound to albumin and α1-acid glycoprotein.^1^ It is generally harmless in small doses; however, rarely, lidocaine is known to cause local anesthetic systemic toxicity (LAST). Lidocaine has a maximum safe dose of 3 mg/kg if used by itself, but the maximum safe dose increases to 7 mg/kg if used in combination with epinephrine.^1^ At levels > 5 μg/mL, patients may begin to experience symptoms such as slurred speech, tinnitus, circumoral paresthesia, and lightheadedness. At levels higher than 10 μg/mL, loss of consciousness and seizures can occur. Beyond 15 μg/mL cardiac arrhythmias, respiratory arrest, and even cardiac arrest can occur.^1^ Other reported signs include muscle twitching, visual and auditory disturbances, dysarthria, agitation, hallucinations, and altered mental status.^2^ Local anesthetic systemic toxicity can also present in a delayed fashion, sometimes up to four days from the initiation of local anesthetic use.^3^

Local anesthetic systemic toxicity symptoms can be progressive when not treated, usually starting with neurologic symptoms and progressing to cardiovascular collapse.^3^ The incidence of LAST is unclear but believed to be low, with an estimate of 0.03%.^4^ In 1997, the incidence of local anesthetic complication during dental procedures in Germany was reported to be 4.5%, although the incidence of severe complications such as seizures was approximately 0.07%.^5^ Despite the rarity of an adverse effect, the rate of lidocaine poisoning and lidocaine mortality has increased since 2010.^6^ Up to 40% of cases have been reported to be atypical, whether it be isolated cardiovascular symptoms, central nervous system symptoms, or significantly delayed presentation.^3^ Thus, knowing the signs and symptoms of LAST including uncommon presentations of an increasingly common toxidrome is important. Only when LAST has been identified can it be treated.

Treatment of lidocaine toxicity at lower levels includes ceasing administration of the drug and observation, while at higher levels 20% intravenous (IV) lipid emulsion therapy can be used.^7^ Ceasing administration of lidocaine is straightforward in situations such as lidocaine infusion, but systemic absorption may be more variable when lidocaine is administered subcutaneously, intradermally, and/or with epinephrine. Therefore, it may need close monitoring or earlier treatment when initial symptoms have been identified. Although the actual mechanism of action of IV lipid emulsion therapy is unclear, it is believed to serve as a “lipid sink” or “lipid shuttle” to remove the local anesthetic in which the fat soluble lidocaine preferentially binds to IV lipid emulsion for transport away from nerves and the heart to organs such as muscle and liver.^8^ Generalized neurotoxicity due to tetracaine injection has been previously reported, with symptoms such as convulsions, rigidity, and aphasia.^9^ However, to our knowledge, lidocaine toxicity presenting as focal neurologic deficits including in the extremities has not been reported prior to now.

## CASE REPORT

A 52-year-old female with past medical history of migraines, cholecystectomy, nephrolithiasis, and hypothyroidism was brought to the emergency department (ED) via ambulance from a dental clinic for acute neurologic complaints and concern for possible stroke. She was receiving an injection of her fifth ampule of lidocaine 2% with 1:100,000 epinephrine intraorally when she suddenly experienced palpitations and left arm and left facial weakness and numbness. She also reported chest heaviness, fatigue, chills, and dizziness but denied headache. Upon arrival to the ED, the patient continued to have left arm and left facial weakness and numbness. Due to the concern for acute ischemic stroke, she underwent computed tomography angiography imaging of the head and neck, which was negative for large vessel occlusions or hemorrhage. An electrocardiogram showed sinus tachycardia with nonspecific ST-segment changes but no prolonged corrected QT interval. Lab studies were unremarkable. There was no leukocytosis or anemia, no metabolic derangements on comprehensive metabolic panel, normal thyroid-stimulating hormone level, and two consecutive normal levels of high-sensitivity troponins. An alternative diagnosis of lidocaine toxicity was considered, prompting administration of 90 mL of 20% IV lipid emulsion therapy. The patient’s left arm and left facial weakness resolved within two hours. She also had residual oral numbness at the site of injection; however, this had resolved by the time she was evaluated by oral and maxillofacial surgery. The patient was cleared and discharged with outpatient follow-up.

*CPC-EM Capsule*

What do we already know about this clinical entity?

*Lidocaine toxicity presents with variable symptoms including slurred speech, tinnitus, seizure, cardiac arrhythmia, and cardiac arrest.*

What makes this presentation of disease reportable?

*Our patient’s symptoms may potentially represent a newly discovered presentation of lidocaine toxicity.*

What is the major learning point?

*Lidocaine toxicity could potentially present as a focal neurologic deficit. Treatment should be considered to prevent disease progression.*

How might this improve emergency medicine practice?

*Given the rarity of lidocaine toxicity, knowing the range of presentations could assist in diagnosis and expediting treatment.*

## DISCUSSION

With the increasing popularity of nerve blocks in many medical specialties, the incidence of LAST is rising. There are many known symptoms; however, given its low incidence it is possible that some symptoms of LAST have not yet been reported. This case involved acute focal neurologic symptoms that began during intraoral lidocaine administration. Given that a quarter of LAST cases present within the first minute of injection, 47% by five minutes, and 67% by 10 minutes, the temporal association between the injection of lidocaine and symptoms in our patient, in addition to the resolution of symptoms after IV lipid emulsion therapy, strongly point toward LAST as the cause of the patient’s symptoms.^2^ Since many hospitals do not have a readily available diagnostic test for LAST (such as a lidocaine concentration assay), coupled with the potential for significant morbidity and mortality if left untreated, it is imperative that treatment be initiated based on clinical suspicion.

We administered 90 mL of 20% IV lipid emulsion therapy and noticed a response within two hours. Focal neurologic findings are typically secondary to stroke, but computed tomography angiography imaging was not suggestive of this diagnosis, and the patient did not report any risk factors typically associated with stroke. Additionally, complex migraine was a possible diagnosis given the patient’s history of migraines, but she did not report any headache to suggest a migraine, nor did she have a history of complex migraines.

There are limitations to this case report. Specifically, a serum lidocaine level was not obtained from the patient. Serum lidocaine level is not an immediately available diagnostic at our institution and, therefore, would not have been helpful in the diagnosis or management in the ED. It may still be worthwhile to obtain a serum lidocaine level as confirmation of the patient’s sensitivity for future treatment considerations. Additionally, five ampules of 2% lidocaine should theoretically result in safe levels of serum lidocaine.

## CONCLUSION

Local anesthetic systemic toxicity is a dose-dependent consequence of lidocaine that can present in a variety of ways with severity of symptoms correlating with the dose given. While some symptoms are well reported, such as slurred speech, tinnitus, circumoral paresthesia, and lightheadedness, the relatively lower frequency of LAST suggests some presentations may not yet have been reported. Given this case, we propose that LAST may present in less common ways such as unilateral facial droop in combination with unilateral arm weakness. This case exemplifies that clinicians should maintain a high index of suspicion for drug-related toxicity, even when presenting symptoms are atypical or not classically associated with the known adverse effect profile of the medication.
